# Research progress on the protective mechanism of a novel soluble epoxide hydrolase inhibitor TPPU on ischemic stroke

**DOI:** 10.3389/fneur.2023.1083972

**Published:** 2023-02-08

**Authors:** Pan Huang

**Affiliations:** Department of Neurology, People's Hospital of Deyang City, Deyang, Sichuan, China

**Keywords:** TPPU, ischemic stroke, blood-brain barrier, ischemia reperfusion, arachidonic acid

## Abstract

Arachidonic Acid (AA) is the precursor of cerebrovascular active substances in the human body, and its metabolites are closely associated with the pathogenesis of cerebrovascular diseases. In recent years, the cytochrome P450 (CYP) metabolic pathway of AA has become a research hotspot. Furthermore, the CYP metabolic pathway of AA is regulated by soluble epoxide hydrolase (sEH). 1-trifluoromethoxyphenyl-3(1-propionylpiperidin-4-yl) urea (TPPU) is a novel sEH inhibitor that exerts cerebrovascular protective activity. This article reviews the mechanism of TPPU's protective effect on ischemic stroke disease.

## 1. Introduction

Ischemic stroke is a serious condition that endangers human health. The prevalence and incidence of stroke in China is rising faster than in other countries due to an aging population, the continued high prevalence of risk factors such as hypertension and diabetes and irregular management. Studies have shown that the prevalence of stroke among Chinese residents aged ≥40 years is 2.58%, or ~17.5 million people. The prevalence of stroke in the adult population (≥18 years) is ~1.29%, with significantly higher increases in the male population and in urban areas (male vs. female: 18.1 vs. 7.3%; urban vs. rural: 18.6 vs. 9.9%), and overall China is facing the greatest stroke challenge in the world (1). Neuroprotective therapy has always been the primary choice in ischemic stroke treatment. In the past 30 years, various neuroprotective agents have been developed for the pathophysiology of cerebral ischemia, including antioxidants, calcium channel antagonists, excitatory amino acid receptor inhibitors, and neurotrophic factors. Experimental research and over 100 clinical studies, most of which are effective in animal experiments but ineffective in clinical trials, have resulted in clinical translation failure (2). Clinical guidelines have not yet suggested an effective neuroprotective agent; however, the exploration of an effective neuroprotective agent in humans continues (3).

**Figure 1:**
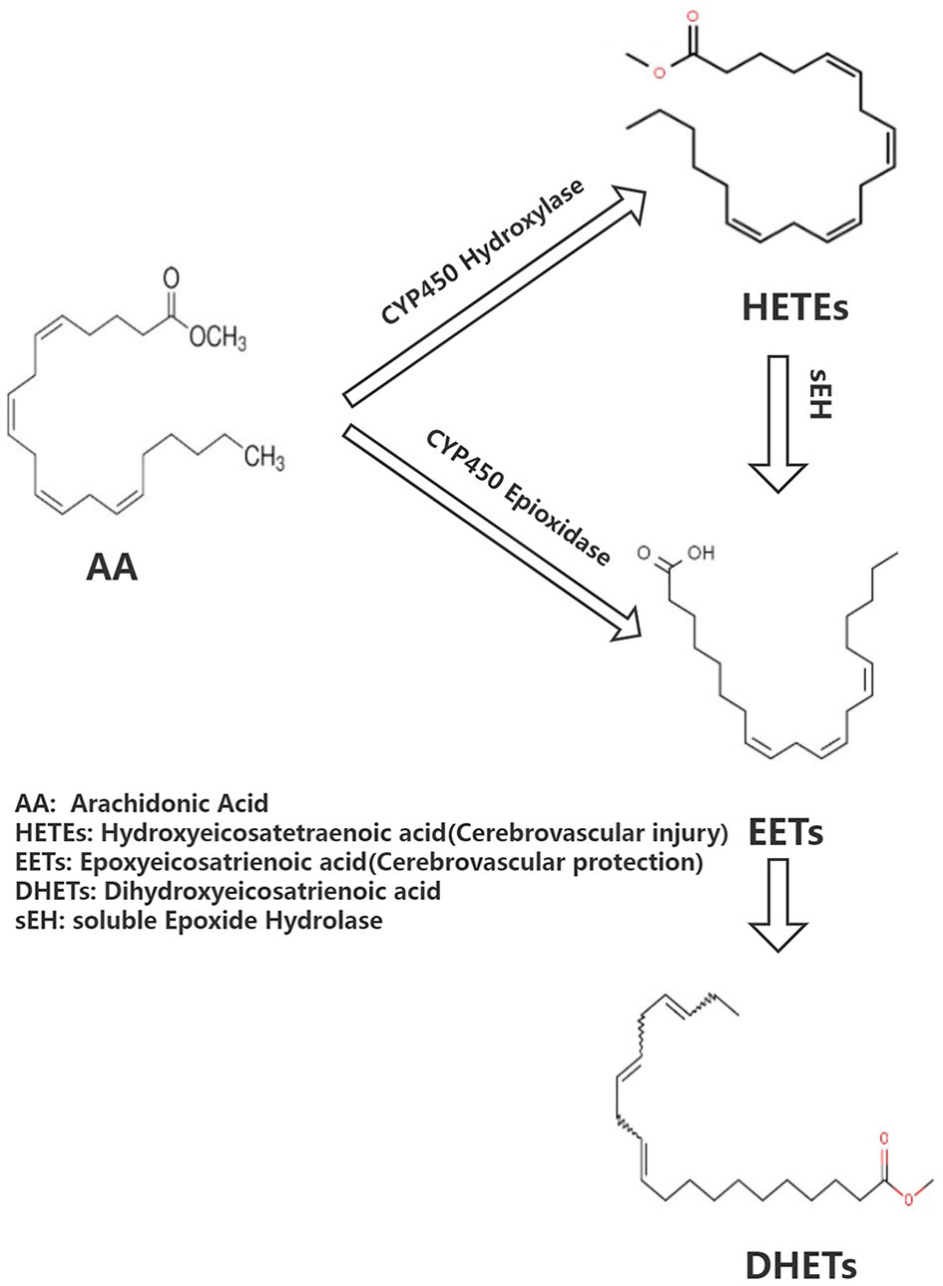
Brief overview of arachidonate acid cascade.

Arachidonic acid (AA) is the precursor of cerebrovascular active substances in the human heart, and its metabolites are closely associated with the pathogenesis of cerebrovascular diseases (4). In recent years, the cytochrome P450 (CYP) metabolic pathway of AA has been a research hotspot (5). AA generates hydroxyeicosatetraenoic acid (HETEs) and epoxyeicosatrienoic acid (EETs) under the action of CYP hydroxylase and CYP epoxidase, respectively. Furthermore, EETs undergo the action of soluble epoxide hydrolase (sEH) to generate dihydroxyeicosatrienoic acids (DHETs) with weak biological activity (Figure 1). HETEs have potent cerebral vasoconstriction and pro-atherosclerotic effects. EETs possess various biological functions, including vasodilation, regulation of ion channels, and anti-atherosclerosis, as well as a protective effect on cardiovascular and cerebrovascular diseases. Previous studies have shown that the levels of CYP metabolic pathway metabolites (EETs and HETEs) of AA are closely associated with the deterioration of neurological function following acute ischemic stroke, implying that they may have a role in cerebral ischemia (6).

sEH is a key rate-limiting enzyme regulating EETs. Previous research has shown that the reduction of peripheral blood EETs is not only closely associated with the deterioration of neurological function after ischemic stroke but also with the degree of carotid artery stenosis and plaque instability in patients with cerebral infarction and is regulated by the gene encoding sEH (epoxide hydrolase 2, EPHX_2_) (7–9). Another study has shown that EPHX_2_ gene knockout can increase cerebral blood flow, reduce infarct volume in rats with middle-arterial artery occlusion, and has a protective effect on cerebral ischemia. sEH is considered a novel target for ischemic stroke prevention and treatment (10). 1-trifluoromethoxyphenyl-3(1-propionylpiperidin-4-yl) urea (TPPU) is a novel sEH inhibitor that exerts a cerebrovascular protective effect. This article includes national and international basic original studies on TPPU intervention in ischaemic stroke, excluding: 1. studies that are more than 20 years old; 2. previous review studies on TPPU for ischaemic stroke. This paper provides a comprehensive understanding of the possible mechanisms of TPPU intervention in ischaemic stroke and hopefully contributes to the search for new targets for the treatment of ischaemic stroke.

## 2. Research status of sEH inhibitors on cerebral ischemia protection

Neuroprotection against sEH has been a hot topic in recent years. Although EPHX2 gene knockout has a protective effect on experimental cerebral ischemia, it is still distant from clinical prevention and treatment of ischemic stroke. Therefore, sEH inhibitors R&D has attracted much attention. In 2005, the she inhibitor 12-(3-adamantan-1-yl-ureido)-dodecanoic acid (AUDA) was applied to the cerebral ischemic nerve. In protective experimental studies, it has been confirmed that AUDA has a protective effect on cerebral ischemia (11). Furthermore, in 2007, she inhibitor, trans-4-[4-(3-adamantan-1-yl-ureido)-cyclohexyloxy]-benzoic acid (t-AUCB), improved the cerebral blood flow and had a protective effect on experimental cerebral ischemia. AUDA and t-AUCB have anti-apoptotic, anti-oxidative, and anti-inflammatory properties, inhibit Ca2+ influx, protect mitochondria, and antagonize N-Methyl-D-Aspartate Receptor (NMDAR)-mediated excitotoxicity, among other mechanisms in brain protection (12). However, these two traditional sEH inhibitors have shown poor absorption and bioavailability from the gastrointestinal tract. The drug dose needed to obtain the effective blood concentration must be substantial enough because the drugs are easily accumulated in the body, the side effects are severe, and animal tolerance is poor. These traditional sEH inhibitors were found to have short half-lives and unstable blood concentrations *in vivo*, resulting in the failure of clinical translation of traditional sEH inhibitors.

## 3. Overview of TPPU

**Figure 2:**
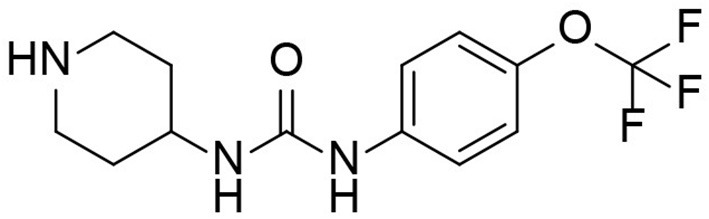
Molecular structure of TPPU.

TPPU is a novel sEH inhibitor synthesized in 2012 by Professor Bruce from the Molecular Bioscience Center of the University of California Veterinary Medicine (13). Bruce Hamock is an entomologist whose research focuses on inhibitors of epoxides in rodents. In his basic research, Professor Bruce found that TPPu has therapeutic and protective effects against various diseases and may be a potential treatment for these diseases. TPPU has a molecular weight of 359.3, allowing it to easily cross the blood-brain barrier and bind to sEH in the central nervous system, inhibiting sEH activity (Figure 2). TPPU has also been proven in animal models of coronary atherosclerotic heart disease to prevent myocardial fibrosis following myocardial infarction and has various functions, including anti-apoptosis, anti-oxidation, and mitochondrial protection (14).

## 4. The protective mechanism of TPPU on stroke

### 4.1. TPPU inhibits inflammatory response after stroke

**Figure 3:**
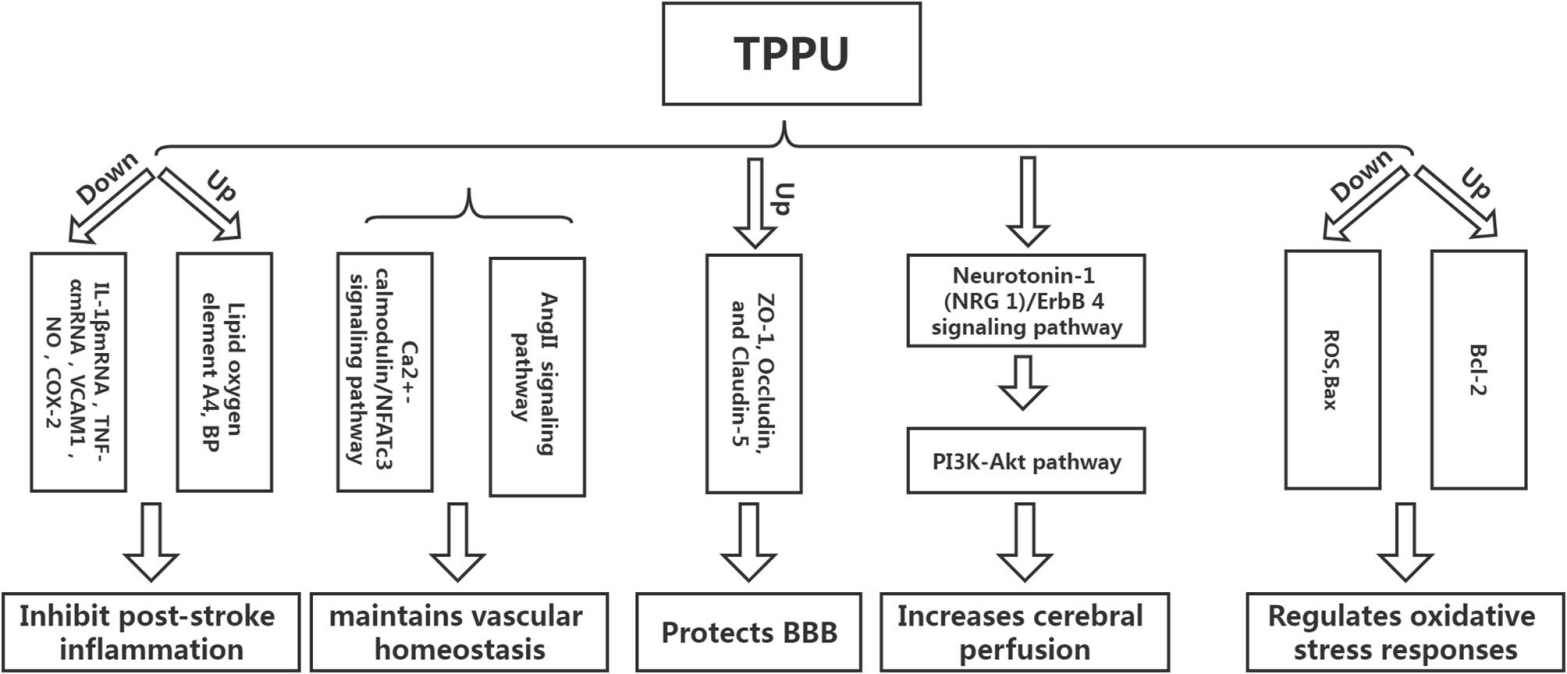
The protective mechanism of TPPU on stroke.

Inflammation can not only cause a cerebral infarction, but it can also further activate the inflammatory response, creating a vicious circle (15–18). Tu et al. developed a mouse model of cerebral infarction and found that TPPU could significantly promote the recovery of neurological function and reduce the infarct volume and the expression of inflammatory cytokines IL-1βmRNA and TNF-αmRNA, revealing that TPPU may reduce the inflammatory response after infarction and promote the recovery of neurological function (19). Yu et al. used endothelial human Nox4 dominant-negative (EDN) transgenic mice in an ApoE deficient background to mimic the dysfunction of endothelial Nox4 in atherosclerosis-prone conditions. sEH and the inflammatory marker vascular cell adhesion molecule 1 (VCAM1) were upregulated in EDN aortic endothelium. TPPU reduced atherosclerotic lesions in EDN mice. In EDN endothelial cells (ECs), the endoplasmic reticulum stress inhibitor, 4-phenyl butyric acid (4-PBA), downregulated the expression of sEH and VCAM1 and suppressed inflammation. Moreover, its application *in vivo* reduced atherosclerotic lesions of EDN mice (20). In a study by Schmelzer et al., lipopolysaccharide (LPS, 10 mg/kg) was injected into C57BL/6 mice to cause celiac inflammation 24 h before and after the subcutaneous TPPU injection (20 mg/kg) and at the same dose of processed palm in the control group. The findings revealed that all the mice in the experimental group survived, and their blood pressure was restored to pre-LPS levels 24 h later, while all the mice in the control group developed severe hypotension (systolic pressure <40 mmHg) and died within 4 days. Studies have shown that TPPU increases the EET indirect inhibition of nitric oxide synthase, reduces NO produce, hypotension due to reduce inflammation, and inhibits non-specific inflammation index COX-2 (Cyclooxygenase-2) expression. Simultaneously, suppressing TNF alpha, IL-6, and monocyte chemotactic factor may reduce inflammation, improve lipid oxygen element A4 production, and increase inflammation abreaction (21). TPPU can simulate the role of EET in hemodynamics and anti-inflammatory activities and regulate growth, utility, and the forecast for new atherosclerosis compounds (22) (Figure 3).

### 4.2. TPPU maintains vascular homeostasis

Homeostasis of vascular structure and function is the basis of human physiological activities. If the vascular homeostasis is unbalanced, it may lead to the occurrence of various diseases (23). Vascular injury is one of the reasons for the imbalance of vascular homeostasis. After cerebral infarction, a vascular injury will inevitably occur, and the adventitia of blood vessels will undergo corresponding changes. Studies have shown that neutrophils can be detected in the adventitia 0.5 h following balloon stretch injury in the coronary arteries of the pig, while the intima-media can be detected later. The vascular endothelial growth factor can be detected in the adventitia at the earliest after balloon-stretch injury to the carotid artery of rats (24). The above findings suggest that the adventitia is the origin and active participant of vascular diseases and is one of the novel targets for treating abnormal vascular function. Protecting adventitia may reduce the imbalance of vascular homeostasis, thereby facilitating disease repair. Angiotensin II inhibitor competes with Ang II for AT1. Furthermore, it inhibits vasoconstriction, promotes aldosterone secretion, reverses hypertrophic cardiomyocytes, and lowers blood pressure. However, AngII-related preparations can result in vascular wall thickening and collagen deposition, affecting the remodeling of the vascular adventitia and causing an imbalance in vascular homeostasis. Researchers have found that TPPU intervention in AngII model mice could significantly prevent AngII-induced vascular adventitia damage. Furthermore, *in vitro* studies have also found that TPPU may affect collagen synthesis *via* the Ca_2+_-calmodulin/NFATc3 signaling pathway, suggesting that TPPU may be one of the novel ways to treat vascular adventitial injury (25).

### 4.3. TPPU protects the blood-brain barrier

Tight-junction proteins between endothelial cells, basement membrane, the foot process of astrocytes, and pericytes are the key structures that maintain the integrity of the blood-brain barrier (BBB). The main pathogenic changes in the early stages of cerebral ischemia are increased BBB permeability and damage of tight junction protein, which causes brain edema, directly or indirectly leading to neurological function damage. Hence, preventing ischemic stroke requires protecting the BBB, inhibiting increased permeability, and alleviating cerebral edema (26). Yi et al. found that TPPU can significantly reduce BBB damage in model rats following cerebral infarction by increasing the expression of ZO-1, Occludin, and Claudin-5, the subunits of tight junction proteins related to BBB (24). Claudin-5 regulates the normal and disturbed states of the BBB. The down-regulation of claudin-5 can directly cause an increase in BBB permeability. ZO-1, a regulator of tight junction proteins, plays an important role in maintaining cytoskeleton formation, cell polarity, and paracellular barrier (27). Multiple studies have reported that tight junction proteins are crucial in regulating BBB integrity and permeability, and the downregulation of tight junction protein expression is associated with increased BBB permeability (28, 29). Yi et al. found that a higher dose of TPPU (2 mg/kg) had a better protective effect on cerebral edema than a lower dose. However, the mechanism may be related to the pharmacokinetics of TPPU, as higher doses did not improve the protective effect (25). TPPU is readily absorbed and slowly eliminated, allowing it to remain in the bloodstream longer than other sEH inhibitors due to its metabolic stability. Previous studies have demonstrated that even at the lowest dose of 0.1 mg/kg, the concentration of TPPU *in vitro* is higher than the IC50 value. When the TPPU dose exceeds 1 mg/kg, the EET/DHET ratio is lower than the TPPU dose of 1 mg/kg, indicating that TPPU should not be overused (13). Furthermore, studies have shown that TPPU can also reduce the damage to the BBB by reducing the damage to vascular endothelial cells. However, there are few studies on this aspect, and the detailed mechanism is unclear (30).

### 4.4. TPPU increases cerebral perfusion

The decrease in perfusion volume after cerebral infarction is the primary reason for the aggravation of neurological function. Low perfusion can not only stimulate the intensification of the local inflammatory response but also result in the activation of excessive oxygen free radicals, causing the aggravation of the disease (31). Hao et al. established a coil-type carotid artery stenosis model. They found that the neurological function of mice was significantly improved after TPPU intervention compared with the control group. Furthermore, basic research has shown that the neuroprotective effect of TPPU on cerebral hypoperfusion may be associated with the activation of the Neuregulin-1 (NRG 1)/ErbB 4 signaling pathway, which can further trigger the PI3K-Akt pathway, implying that TPPU can play a multi-targeted protective effect and reduce the degree of nerve damage in mice with chronic cerebral hypoperfusion (32). However, the increase in cerebral perfusion after cerebral infarction is a reason for the deterioration of neurological function; hence, it is particularly important to explore the appropriate dose of TPPU to achieve a balance between the two. Currently, there are limited reports on TPPU improving ischemic stroke perfusion, and more studies are required to demonstrate the specific mechanism in the future.

### 4.5. TPPU regulates oxidative stress responses

Apoptosis is one of the primary reasons for brain injury after ischemic stroke, and oxidative stress is the main pathway of apoptosis. Studies have shown that TPPU can reduce the production of reactive oxygen species (ROS) after ischemic stroke, increase the expression of Bcl-2 protein, and decrease the expression of Bax protein, implying that TPPU can inhibit cell apoptosis after ischemic stroke (33). ROS production plays a key role in the breakdown of the blood-brain barrier during cerebral ischemia/reperfusion (34). After cerebral ischemia, the expression of ROS and inflammatory cytokines increases, causing mitochondrial damage, activation of pro-apoptotic protein Bax, and stimulation of cytochrome c cascade reaction. Moreover, the Bcl-2 protein can inhibit the downstream apoptotic cascade and block the release of cytochrome C (35).

## 5. TPPU future transformation

Due to the widespread existence of sEH in the human body, its metabolic pathway is involved in many diseases, including ischemic stroke, hypertension, heart disease, kidney disease, etc. In theory, the development of drugs that inhibit sEH would seem to reverse or treat the disease. Human drug exploration for sEH has never stopped. From the early discovery of epoxide sEH inhibitors to the design and synthesis of the third generation of urea human sEH inhibitors, the research on human sEH inhibitors has experienced more than 30 years of development. sEH inhibitors have progressed from the initial sEH inhibitors with only micromolar inhibitory activity *in vitro* and poor or no activity *in vivo* to the current sEH inhibitors with nanomolar activity *in vitro* and remarkable pharmacokinetic properties *in vivo* (36).

TPPU is a novel sEH with good activity *in vivo*. It has been used in basic research in various cardiovascular and cerebrovascular diseases. It can play a protective role through a variety of potential mechanisms. However, it has not yet entered the clinical research stage for cerebrovascular diseases. With the development of pharmacology, the effects and mechanisms of TPPU are constantly being discovered and clarified, further proving its rationality and effectiveness as a treatment for ischemic stroke. As the research progresses, TPPU and its derivatives could be novel drugs for treating ischemic stroke-related diseases.

In conclusion, TPPU can intervene in ischemic stroke in various ways and may be one of the new targets for treating acute ischemic stroke. However, the treatment of acute ischemic stroke with TPPU is still in the exploratory stage, no clinical research has been carried out yet, and more trials are needed to confirm its potential.

## Author contributions

The author confirms being the sole contributor of this work and has approved it for publication.
